# Predatory Abilities of Two Mediterranean Ants on the Eggs and Larvae of the Codling Moth *Cydia pomonella*

**DOI:** 10.3390/insects14020097

**Published:** 2023-01-17

**Authors:** Enrico Schifani, Daniele Giannetti, Donato A. Grasso

**Affiliations:** Department of Chemistry, Life Sciences & Environmental Sustainability, University of Parma, Parco Area delle Scienze, 11/a, 43124 Parma, Italy

**Keywords:** biological control, pest management, Formicidae, Tortricidae, Lepidoptera, *Crematogaster scutellaris*, *Tapinoma magnum*

## Abstract

**Simple Summary:**

Ants are widespread across terrestrial ecosystems, including agroecosystems, where they take part in several important processes. They often can act as predators of a wide range of insect pests in agricultural fields, which should be considered by management programs, and can sometimes be actively exploited to promote sustainable biological control strategies. In a recent experiment conducted in Europe, pear trees visited by larger numbers of ants suffered fewer attacks to their fruits by the codling moth, a small lepidopteran, which is a significant economic pest worldwide, especially in apple, pear, and walnut orchards. However, the exact form of the interaction between the ants and codling moths remained unclear. While ants were already known to prey upon mature larvae or pupae in the soil, this new evidence suggested they could also control the eggs or newly hatched larvae that had not yet attacked the fruits, which are the two stages whose removal would directly prevent fruit damage. We conducted laboratory experiments to determine whether two common European ants could prey upon these stages. Our results suggest that these ants are effectively able to kill newly hatched larvae, while the eggs do not appear directly vulnerable to predation. Further investigation under field conditions would be needed to assess whether ants may also interfere with the oviposition by adult moths.

**Abstract:**

The predatory ability of ants (Hymenoptera, Formicidae) against insect pests can offer an important service to agricultural activities and may sometimes be directly exploited in biological control strategies. The codling moth *Cydia pomonella* (Lepidoptera, Tortricidae) is a major agricultural pest of fruit orchards, whose biological control is complicated by the fact that the larvae spend most of their life protected within the fruits they damage. In a recent experiment in Europe, pear trees in which ant activity was artificially increased by the addition of sugary liquid dispensers (artificial nectaries) suffered less damage caused by the larvae to their fruits. While some ants were already known to prey upon the mature larvae or pupae of *C. pomonella* in the soil, prevention of fruit damage would require predation upon eggs or newly hatched larvae, which have not yet excavated into the fruits. We verified whether two different Mediterranean ants frequently observed in fruit orchards, *Crematogaster scutellaris* and *Tapinoma magnum*, were able to prey upon *C. pomonella* eggs and larvae in laboratory conditions. Our experiments demonstrated that both species similarly attacked and killed young *C. pomonella* larvae. On the other hand, the eggs mostly attracted the attention of *T. magnum* but were never damaged. Further field assessments are required to understand whether ants may also interfere with oviposition by adults or whether larger ant species, although generally rarer in orchards, may also prey upon eggs.

## 1. Introduction

Ants (Hymenoptera, Formicidae) are among the most successful insect groups, and their widespread presence in terrestrial habitats has significant ecological consequences [1,2]. Their relationship with plants is of particular interest from both an evolutionary and an applied perspective [2,3]. One of the most important services that ants may provide to plants in these relationships is protection from a range of different herbivore insects that ants may prey upon or at least displace [4,5]. In addition, ants in agriculture may also play important roles in soil enrichment and bioturbation, as well as control of weeds and certain plant pathogens [6,7,8]. Ants’ ability to protect certain honeydew insect pests must be acknowledged; at the same time, their generalist predatory ability against several phytophagous arthropods promotes their recognition as biological control agents across different agricultural contexts [4,5,9]. This is especially well known in the tropics and comparatively less studied in temperate regions [4].

The codling moth, *Cydia pomonella* (Linnaeus, 1758) (Lepidoptera, Tortricidae), is a key polyphagous fruit pest whose economic relevance is particularly significant in apple, pear, and walnut orchards [10,11,12]. Its control is complicated by the development of resistance against pesticides and baculoviruses [12,13,14], while pesticide usage may disrupt the control of secondary pests [15]. Biological control strategies normally focus on last instar larvae that seek a shelter to pupate, on pupae, or on adults, using predators, parasitoids, and viruses [16,17,18,19,20,21]. In addition, pheromones or the sterile insect technique can be used in mating disruption strategies [22,23,24]. However, few biological control agents are known to target eggs or younger larvae, which spend almost their entire life protected inside the fruit they consume except for a short window after hatching (usually within 24 h), during which they may travel for up to a few meters searching for some fruit to dig into [10,25]. Predatory heteropterans and earwigs are the only known predators of eggs [26,27,28], which are very small (1–1.2 mm long), may be laid directly on the surface of fruits or on nearby areas of the plants, and hatch in about 5–12 days [10,25].

Among the different generalist predators that may play a role in the control of *C. pomonella* [29], ground-dwelling ants can prey upon last instar larvae and pupae [18]. More recently, field data suggested that trees visited more intensively by ants may suffer less damage to their fruits by the moths [30].

While this result suggested an effect of ants on the activity of *C. pomonella* before its larvae dig into the fruits, it remained unclear whether ants affected the eggs and/or the newly hatched larvae [30]. We aimed to test whether two Mediterranean ants that are common in fruit orchards and agroecosystems, *Crematogaster scutellaris* (Olivier, 1792) and *Tapinoma magnum* Mayr, 1861 [31,32], may act as predators of *C. pomonella* eggs and/or newly hatched larvae by documenting their behavioral interactions in laboratory experiments.

## 2. Materials and Methods

All experiments were conducted during June 2022. Four days before the experiments, fragments of *C. scutellaris* and *T. magnum* of at least 500 workers each [33] were taken from Parma University Campus (northern Italy) and temporarily reared under laboratory conditions (T: 25 ± 1 °C, RH: 60 ± 0.5%, photoperiod 12:12 L:D; honey provided as food). Commercially available *C. pomonella* eggs were obtained from Andermatt Biocontrol (Grossdietwil, Switzerland) and kept under the same laboratory conditions. *Cydia pomonella* eggs and first-instar larvae (hatched in the previous 2–8 h) were used in the experiments alongside ant workers randomly selected from the colony fragments. 

In each trial, we introduced into a petri dish (⌀ = 9 cm) either an ant and a group of 6 *C. pomonella* eggs laid on a 1 cm × 1.5 cm paper or an ant and a single *C. pomonella* larva. The *C. pomonella* eggs or larva were initially put at the center of the petri dish and the ant was introduced one minute later. When the ant was introduced, the petri dish was filmed for 10 min using a camera to record the behavioral interactions. Insects used for an experimental trial were not reused in any following trial. A total of 12 trials were conducted for each ant species to study its interaction with *C. pomonella* eggs (n = 24), while 15 trials were conducted to study the interaction of each ant species with *C. pomonella* larvae (n = 30).

The videos were subsequently analyzed using the software, Solomon Coder 19.08.02, to evaluate behavioral interactions. We recorded the following behaviors performed by the ants towards the larvae:

(i) Antennation: the ant touches the eggs/larva with its antennae while slowing or stopping nearby.
(ii) Mandible opening: the ant opens its mandibles in front of the eggs/larva without biting.
(iii) Biting: the ant bites the eggs/larva with its mandibles.
(iv) Chemical attack: the ant uses its chemical repellent to the eggs/larvae (this behavior is performed by applying the venom topically, using the spatulate stinger in *C. scutellaris* and by short distance spraying in *T. magnum*).
(v) Transportation/feeding: the ant starts to feed on the eggs/larva or transport them with its mandibles—this is considered as predation and/or as a proxy of food retrieval to the nest.
(vi) Walking over: the ant walks over eggs/larva.

The frequency of each behavior was recorded in each experimental trial. Biting and transportation/feeding were always displayed together in our observations and were therefore treated as a single behavior for the purpose of statistical analyses. Furthermore, at the end of each trial, we inspected under a stereoscopic microscope whether the eggs appeared damaged and whether the larvae were dead or injured. 

We used a generalized linear model (GLM) followed by Tukey’s post hoc tests to analyze the frequency of the behavioral interactions between the ants and the eggs, according to the identity of the ant species and of the behavior, considering their possible interactions. Differences between the two ant species concerning single behaviors performed were analyzed using Mann–Whitney U tests. We used a GLM with binomial distribution followed by Tukey’s post hoc tests to analyze the frequency of the behavioral interactions between the ants and the larvae, according to the identity of the ant species and of the behavior, considering their possible interactions. Differences between the two ant species for single behaviors performed were then analyzed using the chi-square test. The data were analyzed using the software R 4.2.2 and RStudio [34,35].

## 3. Results

**Figure 1:**
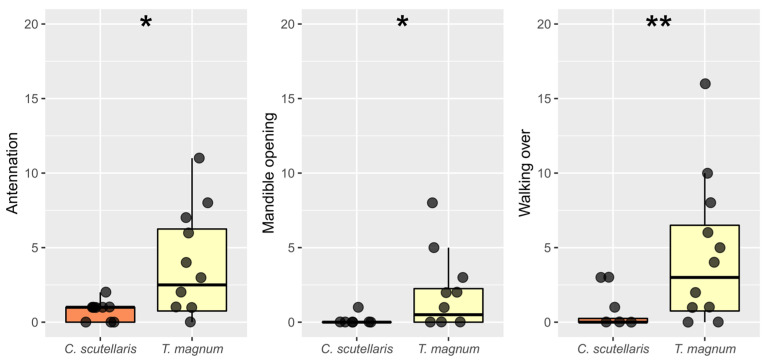
The interactions observed between the ants (*Crematogaster scutellaris* and *Tapinoma magnum*) and the *Cydia pomonella* eggs. Asterisks represent the significance level of the differences between the two species (*, *p* ≤ 0.05; **, 0.001 < *p* ≤ 0.01).

In the interactions between the ants and the eggs, only three behaviors were observed: antennation, mandible opening, and walking over (see Supplementary File S1). We found no significant difference in the frequency of the different behaviors (0.864 ≤ *p ≤* 0.997), while *T. magnum* interacted more frequently with the eggs as compared with *C. scutellaris* (*p* = 0.009). All three behaviors were more frequently expressed by *T. magnum* as compared to *C. scutellaris* (Antennation: W = 37, *p* = 0.038; Mandible opening: W = 39.5, *p* = 0.021; Walking over: W = 29.5, *p* = 0.009; Figure 1, Supplementary File S2). No eggs were harmed by the ants during the trials.

**Figure 2:**
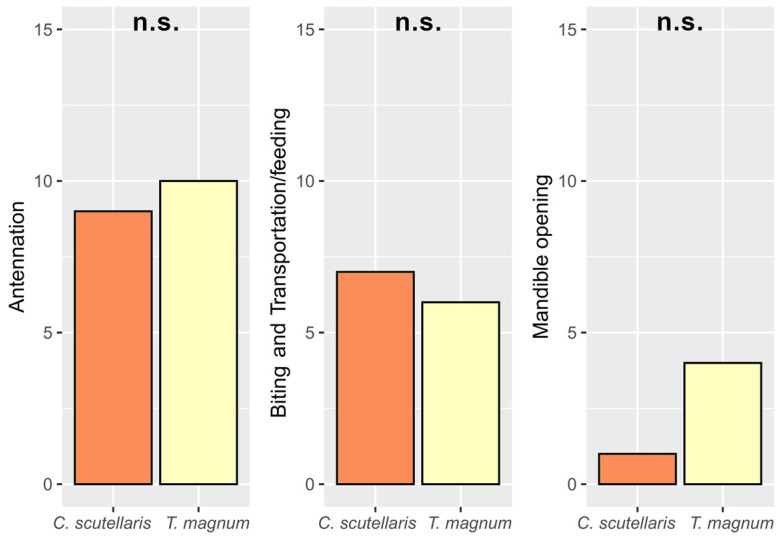
The interactions observed between the ants (*Crematogaster scutellaris* and *Tapinoma magnum*) and the *Cydia pomonella* larvae. No statistically significant differences (n.s.) between the two ant species were detected.

In the interactions between the ants and the larvae, four behaviors were observed: antennation, biting and transportation/feeding, and mandible opening (see Supplementary File S1). Each behavior was observed only once per experiment. Mandible opening was performed significantly less frequently than antennation (*p* = 0.023), while no significant differences were detected between the frequency of the interaction by the two ant species (*p* = 0.705) nor between the frequency of individual behaviors (Antennation: χ^2^ = 0.14, *p* = 0.705; Biting and Transportation/feeding: χ^2^ = 0.13, *p* = 0.712; Mandible opening: χ^2^ = 2.16, *p* = 0.142; Figure 2, Supplementary File S2). Biting and transportation/feeding always implied that the larvae were dead by the end of the experiment; so, 43% of the larvae were killed during the 10-min trials.

## 4. Discussion

Our data revealed that common Mediterranean ants may act as predators of newly hatched *C. pomonella* larvae. Newly hatched larvae are particularly vulnerable to predators, as well as temperature variation and rainfall, until they can locate and excavate into fruit, which may take from 10 min to a few hours to accomplish [25,36]. In our experiments, both *C. scutellaris* and *T. magnum* behaved similarly towards the larvae, killing them in approximately half of the short trials by repeatedly biting their soft parts and then immediately feeding on them or transporting them with their mandibles. Detection through antennation was typically followed by attacks, while in most trials in which no attacks were recorded, the larvae remained undetected. We can speculate that very small newly hatched larvae may be a more attractive and more easily encountered item for smaller ants. While both species did not attack the eggs, these attracted the attention of *T. magnum* significantly, as the workers were repeatedly observed performing stereotyped mandible threats and often kept antennating or walking over them several times. Eggs may offer little foothold to the ants’ mandibles and can adhere strongly to the substratum of leaves and fruits, thus becoming physically invulnerable at least to the species we tested [37,38]. Larger ants with stronger and larger mandibles may be more capable of damaging or feeding on the eggs, but they are often less frequent in agroecosystems [32]. While we cannot entirely discard other possible mechanisms of protection (e.g., chemical repellency or insignificancy), the eggs appeared to be attractive for ants during our experiments (especially in the case of *T. magnum*), which may at least increase the chances that ant workers can take advantage of the moment they hatch to prey upon the larvae. In our experiment, whenever the ants attempted to attack a larva, the larva was always successfully killed. However, even if young larvae manage to escape ants, any delay in their effort to find and excavate fruit is expected to result in significantly higher mortality rates [36]. Based on the evidence of other ant–plant–phytophagous interactions, it is also possible that the excavation behavior by the young larvae releases semiochemicals that are attractive to ants [39].

Both ant species we used in our experiments are known to be able to act as predators of many other agricultural pest insects [31,40,41,42]. Potentially problematic relationships with aphids or coccids are also possible in some cases [43,44]. Manipulation of nesting site availability and trophic resource may be crucial to maximizing the benefits of these ants in biological control strategies [30,32]. Further efforts should focus on interactions between *C. pomonella* and ants in the field [18,30]. For instance, potential interference between ants and ovideposing adults has so far not been investigated but may contribute to explaining the reduction in damaged fruits in ant-visited plants [30]. In fact, in several ant species, more or less specialized workers may function as a constant “presidium”, exploring and patrolling even large areas in search for suitable resources [1,45,46].

If predation of *C. pomonella* larvae in the field is confirmed to be significant, it is possible that adult moths prefer to avoid laying their eggs in ant-visited fruits even without coming into direct contact with the ants, as observed in similar interactions with fruit flies or scolytid beetles, which are mediated by semiochemicals [42,47]. In fact, it is well known that, apart from chemical trails, both arboreal and ground-dwelling ants may lay additional markers on patrolled and defended areas [48,49,50]. 

In conclusion, the predatory role of ants in temperate agroecosystems is for the most part still little understood [30,31,51,52], but due to their ubiquitous presence and generalist feeding habits, ants are likely to play a significant yet overlooked role in the control of the populations of several pest insects.

## Data Availability

The data presented in this study are available in the Supplementary Materials.

## Supplementary Materials

The following supporting information can be downloaded at: https://www.mdpi.com/article/10.3390/insects14020097/s1, Supplementary File S1: Video documentation of behavioral interactions; Supplementary File S2: Behavioral data and GLM output.
